# Allele-specific genome targeting in the development of precision medicine

**DOI:** 10.7150/thno.43298

**Published:** 2020-02-10

**Authors:** Junjiao Wu, Beisha Tang, Yu Tang

**Affiliations:** 1 National Clinical Research Center for Geriatric Disorders, Department of Geriatrics, Xiangya Hospital, Central South University, Changsha, Hunan 410008, China.; 2 Department of Rheumatology and Immunology, Xiangya Hospital, Central South University, Changsha, Hunan 410008, China; 3 Department of Neurology, Xiangya Hospital, Central South University, Changsha, Hunan 410008, China; 4 Key Laboratory of Hunan Province in Neurodegenerative Disorders, Central South University, Changsha, Hunan 410008, China

**Keywords:** genomic editing, CRISPR, genetic variants, SNP, allele-specific

## Abstract

The CRISPR-based genome editing holds immense potential to fix disease-causing mutations, however, must also handle substantial natural genetic variations between individuals. Previous studies have shown that mismatches between the single guide RNA (sgRNA) and genomic DNA may negatively impact sgRNA efficiencies and lead to imprecise specificity prediction. Hence, the genetic variations bring about a great challenge for designing platinum sgRNAs in large human populations. However, they also provide a promising entry for designing allele-specific sgRNAs for the treatment of each individual. The CRISPR system is rather specific, with the potential ability to discriminate between similar alleles, even based on a single nucleotide difference. Genetic variants contribute to the discrimination capabilities, once they generate a novel protospacer adjacent motif (PAM) site or locate in the seed region near an available PAM. Therefore, it can be leveraged to establish allele-specific targeting in numerous dominant human disorders, by selectively ablating the deleterious alleles. So far, allele-specific CRISPR has been increasingly implemented not only in treating dominantly inherited diseases, but also in research areas such as genome imprinting, haploinsufficiency, spatiotemporal loci imaging and immunocompatible manipulations. In this review, we will describe the working principles of allele-specific genome manipulations by virtue of expanding engineering tools of CRISPR. And then we will review new advances in the versatile applications of allele-specific CRISPR targeting in treating human genetic diseases, as well as in a series of other interesting research areas. Lastly, we will discuss their potential therapeutic utilities and considerations in the era of precision medicine.

## Introduction

Inherited human diseases are caused by different types of gene mutations, insertions/deletions (indels), genomic structural variations, as well as pathogenic single nucleotide polymorphisms (SNPs) that are tightly associated with higher risks of diseases. Among those, dominant diseases present a great challenge for conducting gene therapies. Those patients inherited one pathogenic allele causing a disease phenotype, especially in a dominant-negative manner, and one normal allele as well. The treatment strategy thus typically involves the silence of the pathogenic alleles in an allele-specific manner, without affecting the wild-type ones.

During recent years, clustered regularly interspaced short palindromic repeats (CRISPR) has emerged to be a promising tool to treat human genetic diseases by disrupting deleterious gene sites. Basically, the CRISPR system is rather specific, with the potential to discriminate between similar alleles, even based on a single nucleotide difference. Therefore, it can be leveraged to establish allele-specific targeting of those heterogenous alleles.

So far, allele-specific CRISPR has been increasingly utilized not only in treating dominant negative diseases, but also in other multiple areas such as genome imprinting, haploinsufficiency, spatiotemporal loci imaging and immunocompatible manipulations. In this review, we will mainly focus on the versatile applications of allele-specific genome manipulations by virtue of the expanding toolbox of the CRISPR system, and further discuss their potential utilities and considerations in implementing precision medicine.

## Genetic Diseases and Precision Medicine

A genetic disease is caused in whole or in part by an abnormality in the genetic makeup of an individual's genome. Those genetic abnormalities basically involve single-base mutations, indel mutations, duplication mutations, nucleotide repeat expansions and also chromosome structural abnormalities. In the human genome, the most frequent variations are SNPs and short indels 1-3. The average human genome contains roughly 4-5 million SNPs that occur on average every 1000-2000 nucleotides 4, which presents at sufficient density for comprehensive haplotype analysis. Moreover, the recently released Exome Aggregation Consortium (ExAC) data set, with variants from 60,706 individuals, contains approximately one variant for every 8 nucleotides in the human exome 5.

Human genomes have now been sequenced at unprecedented rates with next-generation sequencing technologies, and the era of precision medicine is rapidly approaching. Over the last decade, the emerging next-generation sequencing has facilitated high-throughput genome-wide association studies (GAWS) that have identified numerous pathogenic SNPs 6, 7. Notably, those pathogenic SNPs are by large kinds of gene mutations resided in both coding regions and regulatory regions, and also usually located at functional non-coding RNAs that participated in disease pathogenesis 8-11. SNPs also underline differences in the susceptibility to a wide range of diseases and pharmacological treatment, that may help to guide the personalized drug usage 12-15. Therefore, targeting variants like SNPs may provide a promising entry for silencing deleterious alleles in the era of precision medicine.

## Genome Targeting and CRISPR

Although the concept of gene therapy emerged decades ago, it has been slow to achieve its full potential. This is partly due to concerns related to early clinical trials and the difficulty of safe and accurate targeting. However, with the advent of precise genome editing techniques such as CRISPR, we are on the verge of a gene therapy revolution. The CRISPR/Cas system was initially discovered as the adaptive immune system of the bacterial species by incorporating DNA of invaded phages or viruses into the host genome, which will be stored in an array in DNA 16-18. And later this will be used to cleave the same invaded foreign DNA loci, based on complementarity to the encoded sequence. Over the past years, the CRISPR/Cas system has been extensively developed for targeting genes in mammalian cells 19, 20, and further transformed for curing genetic diseases and engineering desirable genetic traits.

**Figure 1:**
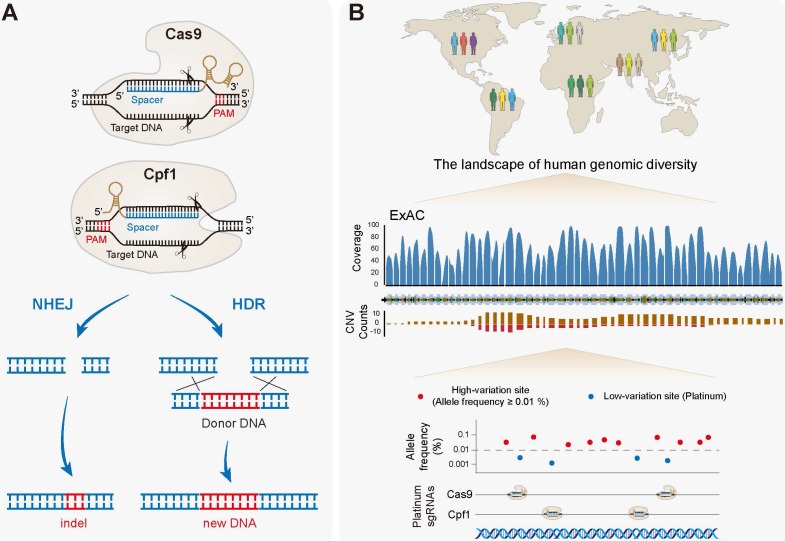
** The CRISPR/Cas system and identification of platinum targets.** (**A**) The basic working principle of Cas9 (Class 2 type II) and Cpf1 (Class 2 type V). An sgRNA/crRNA (brown) encoding a spacer (blue) is bound to a target double strand DNA (black) proximal to a PAM site (red). Perfect base-pairing then activates the nuclease activity, cleaving both DNA strands (scissors) to form DSBs, which are later repaired either by the NHEJ pathway or the HDR pathway. (**B**) The CRISPR-based therapeutic genome editing must also handle with substantial natural genetic variations between individuals. Genetic variants may a great impact on sgRNA efficiencies, as well as both on- and off-target specificities. For the CRISPR-based therapy in large patient populations, genetic variations should be carefully taken into account in designing and evaluating sgRNAs. The solution would be identifying universal sgRNAs located in the low-variation regions. By searching the ExAC browser, for example, people can figure out exome-wide target sites, which lack variants occurring at allele frequencies of ≥ 0.01% (platinum targets). Selection of platinum sgRNAs should maximize the population efficacy of genome editing. The human *LRRK2* gene was viewed as an example.

Basically, the CRISPR/Cas system consists of two components, Cas nucleases and single guide RNA (sgRNA), which form as a complex to bind and cleave double strand DNA (dsDNA) **(Figure 1A)**. Cas binds to a DNA sequence complementary to the sgRNA spacer adjacent to a protospacer adjacent motif (PAM) site. Upon correct base pairing, Cas then activates its nuclease to cleave the dsDNA to form double-strand breaks (DSBs), which are later repaired either by non-homologous end joining (NHEJ) pathway or homology-directed repair (HDR) **(Figure 1A)**. NHEJ results in random indels near the cleavage site that may cause frameshift mutations and premature stops. HDR typically occurs at lower and substantially more variable frequencies than NHEJ, but can be employed to introduce a specific DNA donor precisely at the target site.

Different types of Cas proteins have been discovered with distinct PAM recognition sites. So far, four major types of Cas nucleases including Cas9, Cpf1 (Cas12a), Cas12b (C2c1) and CasX (Cas12e) have been demonstrated to possess DNA targeting activities **(Table 1)**, and available Cas nucleases from other bacterial species have been increasingly characterized 21-32. Types of Cas nucleases possess different protein sizes, unique PAM constraints, cleavage patterns, as well as different lengths of seed regions that may determine targeting specificities (**Table 1**). Currently, the first- and best-characterized CRISPR system is that of Cas9 from *Streptococcus pyogenes* (SpCas9) 33, 34. It requires at least one G in their PAMs, but is quite a large protein containing more than 1300 amino acids, which greatly hinders its usage in the package into adeno-associated virus (AAV) vectors for gene therapy **(Table 1)**. The Cas9 from *Staphylococcus aureus* (SaCas9) 35 was then characterized with the advantage of its smaller size (with ~1000 amino acids), which is suitable for the AAV package. Similarly, other compact Cas9 orthologs, such as NmCas9 (1082 amino acids) 23 and CjCas9 (984 amino acids) 29, can be packaged in all-in-one AAV vectors for *in vivo* editings. Later discovered Cas nucleases such as Cpf1 36-41, Cas12b 42, 43 and CasX 44 are with T-rich PAMs, and thus have broadened the range of genome editings, and are particularly useful in targeting AT-rich genomes or regions. Notably, the recently characterized Cas12b 42, 43 and CasX 44, 45 show to be with quite higher specificities, and also with smaller sizes. To further broaden the available targeting capabilities, Cas nucleases have been continuously engineered as multiple variants for either improved specificity (such as SpCas9-HF 46, eSpCas9 47, HypaCas9 48, SaCas9-HF 49 and enAsCpf1-HF1 50), or expanded available PAM sites 46, 50-58 **(Table 1)**.

In the practice of genome engineering, the CRISPR system has been versatile in multiple areas. Initially it was used to disrupt gene expression by introducing DSBs, no matter with single sgRNAs at coding regions, or dual flanking sgRNAs at coding or non-coding regions. Later on, it was used to delete a large DNA fragment 59, 60, or even one deleterious chromosome by multiple sgRNAs that may be useful for treating diseases like Turner syndrome and Down syndrome 61.

As part of expanded applications, the Cas nuclease was also engineered to be catalytically inactive (dead Cas; dCas) that silenced its cleavage activity without compromising DNA binding activity 36, 62. Due to this unique property, dCas could then be serving as programmable nucleic acid binding scaffolds for the recruitment of a variety of effector proteins/domains that may facilitate chromatin imaging 63, 64, purification of specific genomic loci 65, 66 and proximity labeling 67, 68, epigenetically gene activation 69-72 and gene repression 73, 74, or more excitingly, the base editing 75, 76. Since the expanded engineered Cas variants or expanded tool of the CRISPR system, as well as their respective applications have been recently comprehensively reviewed elsewhere (please refer to 77-84), we are not going to describe them in much more details in this review.

## Genetic Variations and CRISPR-based Therapeutics

The CRISPR-based genome editing holds immense potential to fix disease-causing mutations, however, must also handle with substantial natural genetic variations between individuals. The designed sgRNA based on the reference genome may introduce mismatches between the guide RNA and the genomic DNA from each individual, which will negatively impact sgRNA efficiencies and lead to imprecise specificity prediction 85. Further studies have conducted a more comprehensive analysis of the datasets of ExAC and 1000 Genomes Project (1000GP), and showed that genetic variations may significantly affect the sgRNA efficiencies, as well as both on- and off-target specificities at therapeutic sites 86, 87. Therefore, genetic variants should be carefully assessed in the design and evaluation of sgRNAs for CRISPR-based treatments with large patient populations, otherwise, it may confound clinical trials and also lead to adverse consequences. The key would be identifying universal sgRNAs located in the low-variation regions 86. For instance, by searching the ExAC browser 88, or the recent Genome Aggregation Database (gnomAD) browser 89, people can figure out exome-wide target sites, which lack variants occurring at allele frequencies of ≥ 0.01% (called "platinum" targets) **(Figure 1B)**. Selection of platinum targets should maximize the population efficacy, and even facilitate the therapeutics for super populations of patients with specific variants, based on the demographic information provided by 1000GP 86.

Although the genetic variations would be a challenge for designing platinum sgRNAs in large populations, they may provide a promising entry for designing allele-specific sgRNAs for the treatment of each individual, taking advantage of heterozygous variants. Therefore, the exploitation of genetic variations brings about a concept of “seeking common ground while reserving differences” that not only helps to select the universal platinum targets for large populations, but also facilitates the design of unique targets in individuals for precision medicine.

## Allele-specific Genome Targeting

The notion of “allele-specific intervention” has been originally raised for treating dominant diseases 90. Those patients inherited a pathogenic allele and a normal allele on the chromosome pairs. As caused by harmful gain-of-function mutations, gene augmentation is unlikely to effectively alleviate the disease phenotype. The treatment strategy thus typically includes an allele-specific intervention by silencing or ablation of pathogenic alleles without any abnormal effect on wild-type alleles, especially in cases where wild-type allele expression is pivotal for cellular survival. Compared to the conventional editing of both alleles, the sustained expression of healthy alleles will minimize the potential negative effects on human physiology, prevent disease progression, and may even allow for the recovery of normal phenotypes.

It has to be noted that the allele-specific intervention has been widely achieved in the last decade by using either short-interfering RNAs (siRNAs) 91-101 or antisense oligonucleotides (ASOs) 102-106. This potential of allele-specific siRNAs depends on its highly sequence-specific knockdown manner. The imperfect complementarity of siRNAs with mRNAs gives rise to the discrimination ability of suppressing either allele 107, 108. Numerous studies have been successfully conducted to assess the potency and specificity of allele-specific gene suppression for various disorders including skin disorders 97, 98, multiple neurodegenerative diseases 99-105 and retinitis pigmentosa 106, among others, and produced immense therapeutic benefits.

In recent years, allele-specific CRISPR genome editing has emerged to be a potential approach to treat those dominant diseases, although the application of allele-specific Transcription Activator-Like Effector Nucleases (TALEN) has also been reported 109. Similarly, the CRISPR system provides a highly specific genome editing capable of distinguishing pathogenic alleles from wild-type ones, partly based on the imperfect complementarity of sgRNAs with wild-type sequences. However, the differences, or particularly the advantages, of allele-specific CRISPR over siRNAs would be (i) the possibilities of novel PAMs derived from genetic variants, and (ii) the capabilities of targeting any gene locus of interest rather than merely transcribed mRNAs.

Based on the properties of discriminating sgRNAs, allele-specific CRISPR is intended to be used for targeting multiple types of genetic variants, including (i) Single-base mutations and SNPs; (ii) Short indels and nucleotides gaps; (iii) Reversed fragments; (iv) Virus integration (or other element integration); and (v) Chromosome-specific genomic indels.

## Working Models of Allele-specific CRISPR

**Figure 2:**
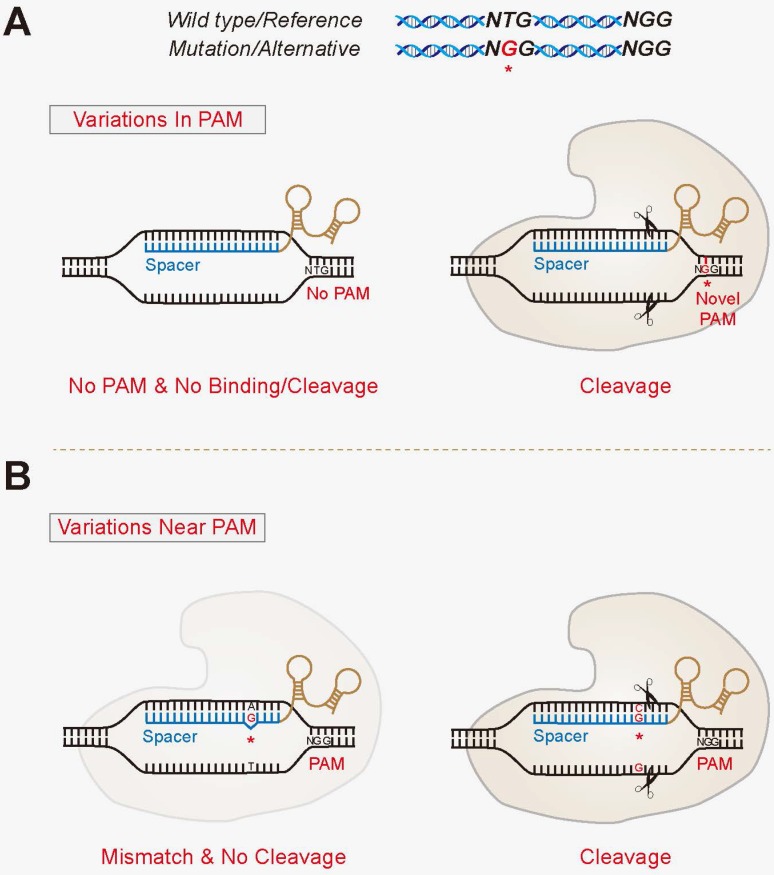
** The proposed model for allele-specific genome targeting by CRISPR.** The “In PAM” model (**A**) and “Near PAM” model (**B**) for allele-specific genomic targeting. The “In PAM” model confers the most stringent allele-specific cleavages, since the binding of Cas with its target DNA is diminished. The “Near PAM” model exploits the discrimination ability of sgRNAs, with mutations or SNPs that locate within the spacer region, particularly the seed region. The variant is denoted by a red asterisk.

Allele-specific CRISPR works largely depending on the structures of discriminating sgRNAs, which contain several critical elements such as PAM sites and spacer sequences that provide the entry points for Cas binding. Genetic variants may present on both PAM and spacers, manipulation of which can be exploited for allele-specific genome targeting. Accordingly, there are basically two types of working models:

**(1) The “In PAM” model.** Gene mutations or SNPs that can create novel PAM sites at only one allele **(Figure 2A)**. And vice versa, they may also eliminate PAM sites at either allele that renders discrimination ability. This model confers the most stringent allele-specific cleavages, since the binding of Cas with target DNA may not even happen without matching PAMs. Exploiting the various PAM sites recognized by different Cas variants produces alterative choices for the allele-specific strategy. For example, SpCas9 recognize a classical 5'-NGG-3' PAM, in which GG would allow for allele specificity, whereas SpCas9-VRER, one of its variants, has a stringent selectivity for a 5'-NGCG-3' PAM site. Interestingly, Scott et al. catalogued variants present among all possible targets in human reference exome using ExAC datasets, and observed that the total ratios of targets containing “In PAM” variants was similar (roughly 21-35%) for SpCas9, SpCas9-VQR, SaCas9 and AsCpf1, whereas 80% of targets were affected by “In PAM” variants for SpCas9-VRER 86. This is due to the fact that the PAM for SpCas9-VRER contains a CpG motif that has been shown to be highly mutable in human exomes 5. Thus, Cas enzymes using PAMs containing CG motifs might be considerably more flexible for allele-specific targeting.

**(2) The “Near PAM” model.** Many pathogenic alleles, however, do not contain mutations that generate novel PAM sites, making them refractory to the “In PAM” model. One potential approach of expanding the applicability is to develop strategies in which the gene mutations or SNPs locate within the spacer region, particularly the seed region, of the discriminating sgRNAs **(Figure 2B)**. Importantly, the seed sequence confers a rather stringent specificity of CRISPR. Studies have demonstrated that base pair mismatches between sgRNAs and their target sequences could significantly reduce or even abolish the efficacy of CRISPR-mediated editing 33, 34. Cong et al. have previously reported that even single-base mismatches in the PAM-proximal seed region can eliminate genome cleavage of SpCas9 19. Also as previously demonstrated by Smith et al. in human induced pluripotent stem cells (iPSCs) and Yoshimi et al. in animal models, merely changing one single nucleotide within sgRNA spacers renders it unable to bind and cleave the target DNA 110, 111. Similarly, in plants, Zhou et al. examined a total of 717 sequences harboring one SNP in each allele near/within the PAM, both of which completely abolished the editing events by the discriminating sgRNAs 112. This feature could thus be exploited to specifically target disease- causing single mutations or SNPs to disable the mutant alleles, whilst insulating against genome modification in the wild-type counterparts.

Different types of Cas nucleases are basically endowed with different lengths of seed regions, which must be taken into account when practicing allele-specific targeting. For instance, the targeting specificity of SpCas9 is largely determined by the 10-12 nucleotides of proximal seeds of 3' PAMs 33; whereas Cpf1 nucleases possess much shorter seeds with 5-6 nucleotides downstream of 5' PAMs 36, 38. Interestingly, another two nucleases, Cas12b and CasX, appear to possess with much longer seed sequences and thus render higher targeting specificities 42-44.

Notably, a comprehensive analysis was recently performed on the genome-wide variants from more than 2500 individuals from the 1000GP and exome variants from more than 60,000 individuals in the ExAC 113. The results showed that most variants reside in or near a PAM site that could affect sgRNA sites recognized by at least one of 11 chosen Cas types 113, which pretty well underpins the practicability of allele-specific targeting.

## Applications of Allele-specific CRISPR

**Figure 3:**
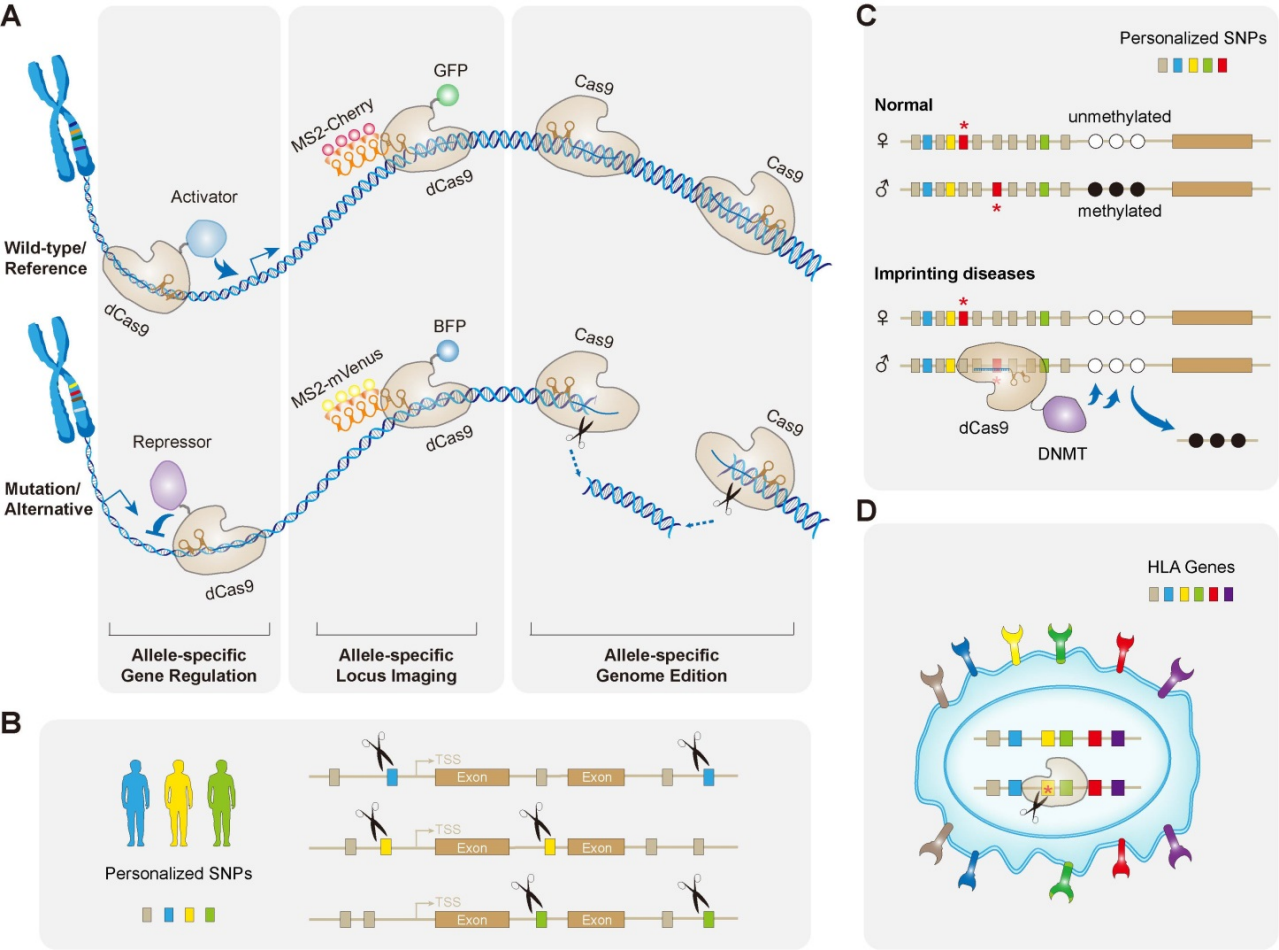
** Major applications of allele-specific genome editing by CRISPR.** (**A**) Apart from allele-specific gene knockout by wild-type Cas through its DNA cleavage activity, catalytically inactive dCas9 fused with various effector proteins/domains has been repurposed to achieve allele-specific loci imaging, gene activation, and gene repression. (**B**) Common SNPs representing a given haplotype can all be used for allele-specific targeting in the era of precision medicine. This strategy uses pairs of custom-designed haplotype-specific sgRNAs, which may permanently ablate any gain-of-function mutations in the human genome. (**C**) Scheme of the concept of correcting imprinting diseases by allele-specific epigenome editing. Normally, paternal and maternal alleles show different DNA methylation patterns (indicated by open and filled circles) at imprinted loci, however, they are lost in imprinting disorders. This epimutation could be corrected in an allele-specific manner, discriminating by the polymorphisms (denoted by red asterisks). The paternal allele showed here is selectively bound by the dCas9 fused with DNA methyltransferase DNMT that may restore the DNA methylation of this allele. (**D**) Allele-specific CRISPR manipulates the immunocompatibility by selectively targeting HLA haplotype. This strategy could greatly increase donor compatibility for regenerative medicine, and is anticipated to serve more pseudo-homozygous cells donors including iPSCs.

As it was mentioned earlier, allele-specific CRISPR works not only on gene mutations, but also on common or rare SNPs, exploiting of which contributes to its versatile applications **(Figure 3)**. For example, by applying an allele-specific strategy, disease loci with mutations could be successfully modified to create isogenic iPSC lines derived from patients with frontotemporal dementia (FTD), for better disease modeling and cell therapy 114. Similarly, allele-specific targeting was used to create animal models in several studies 115, 116. In the zebrafish model, the polymorphisms were utilized to create deletions on specific chromosomes, and enhance the efficiency of a chromosome-specific loxP site in cis 116. This allele-specific strategy may hence improve the utility of the zebrafish model for genetic studies.

Interestingly, a recent study developed an allele-specific CRISPR live-cell DNA imaging technique (termed SNP-CLING) that is able to resolve allelic positioning relative to nuclear sub-compartments and allele-specific interactions between non-homologous chromosomes 117. To visualize each allele for a given locus simultaneously in living cells, they leveraged dCas9 with two to three sgRNAs by targeting possible SNPs genome-wide in PAM motifs. Briefly, they appended sgRNAs with RNA-aptamer motifs (such as MS2 and PP7) and co-transfected with their corresponding RNA-binding proteins fused to a fluorescent protein (such as mVenus and mCherry), for allele-specific labeling 117. They particularly applied SNP-CLING to two long noncoding RNA (lncRNA) loci, Firre and CISTR-ACT, which are associated with heterozygous structural aberrations on higher-order nuclear architecture that eventually cause Mendelian diseases 117. The technique of SNP-CLING thus overcomes the limitations of previous imaging and chromatin capture techniques in resolving allele-specific spatiotemporal properties of genomic loci in living cells. Importantly, using heterozygous SNPs in haplotypes of human pedigrees may render this imaging technique to be widely applicable to the study of gene locations at the allele-specific resolution **(Figure 3A)**.

In the following part, we will mainly focus on the major applications of allele-specific CRISPR in the field of gene therapies **(Table 2)**.

### Allele-specific CRISPR and Dominant Disorders

For dominant genetic diseases, only one allele harboring a mutation can cause disease phenotypes. Thus, the specific silencing of the disease-causing allele would be therapeutically desirable. Basically there are three scenarios that may provoke autosomal-dominant phenotypes: (i) Haploinsufficiency: a situation in which individuals whose mutations are heterozygous at a particular locus, are clinically affected because a single copy of the normal allele does not provide sufficient protein production to maintain normal functions 5, 118; (ii) Dominant negative effect: a nonfunctional mutant whose expression adversely affects the function of the normal gene 119; (iii) Gain-of-function mutation: a type of mutation that alters the gene product to acquire new abnormal functions that eventually lead to the phenotype 120. As you may also read with details described below, allele-specific CRISPR can actually work well for each scenario to ameliorate the disease phenotypes: (i) by treating haploinsufficiency with selectively activation of wild-type allele expression; or (ii) by effectively disrupting the deleterious alleles with dominant negative effects or with gain-of-abnormal-functions.

So far, retinitis pigmentosa, a group of related eye disorders that cause progressive vision loss, was most treated by allele-specific targeting **(Table 2)**. Mutations in the Rhodopsin (*RHO*) gene are the most common cause of autosomal dominant retinitis pigmentosa (adRP), accounting for 30 to 40 percent of all cases 121. Several pieces of studies have converged to focus on the *RHO* mutations both *in vitro* and *in vivo* 122-125. Burnight et al. first utilized SaCas9 and delivered discriminating sgRNAs **(“Near PAM”)** to iPSCs derived from a patient with the RHO P23H genotype, and observed that modifications only occurred in the mutant allele among the 86 clones sequenced, causing a frameshift and premature stop that would prevent transcription 122. Later, they also packaged the sgRNA-SaCas9 cassette into AAV5, which was injected sub-retinally into a transgenic pig model that carries the human P23H mutation. Similarly, no indels were found in the wild-type counterpart 122. Other studies led by Giannelli et al. and Li et al. alternatively chose the engineered variant SaCas9-KKH, SpCas9-VQR and SpCas9-VRQR for treating retinitis pigmentosa in murine RHO^+/P23H^ mutant retinae, by the intravitreal AAV9-based delivery 123. They tested several discriminating sgRNAs, although some of which did not really discriminate, photoreceptor cell degenerations were successfully slowed down by certain working sgRNAs 124. Those divergent therapeutic results also remind us of the importance of selecting optimal Cas proteins and their matched sgRNAs.

Allele-specific CRISPR was also applied in the treatment of cancers 126-128 **(Table 2)**. One of gain-of-function mutations in oncogene *KRAS* leads to the drug resistance to the MEK inhibitor AZD6244, which plays a critical role in anti-proliferation and pro-apoptosis in various tumor cell lines 129. Allele-specific targeting by SpCas9 was then employed taking advantage of a novel PAM site **(“In PAM”)** created by the heterozygous G13A mutation (GGC > GCC). It come out that the G13A mutation was selectively and completely silenced in colorectal cancer cells, resulting in the reversal of drug resistance 126. Around 15% of non-small cell lung cancer (NSCLC) cases are linked to mutations in the oncogene *EGFR*, which contributes to tumor progression 127. Similarly, Koo et al., targeted *EGFR* harboring a single-nucleotide missense mutation L858R (CTG > CGG) that creates a novel PAM **(“In PAM”)** recognized by SpCas9. The combination of adenovirus delivery of SpCas9 and mutation-specific sgRNA resulted in precise destruction of the oncogenic allele with high specificity, and further promoted the killing of cancer cells and the reduction of tumor size in a xenograft mouse model 127. Those results suggest that selective targeting of oncogenic mutations using CRISPR provides a robust surgical strategy to treat cancers.

Recently, another two excellent work have focused on the therapy of dominant progressive hearing loss. Gao et al. designed and validated that allele-specific editing preferentially disrupted the dominant deafness-related allele in the Tmc1 Beethoven mouse model, although the mutant Tmc1 allele differs from the wild-type allele at only one base pair 130.

Injection of SpCas9-sgRNA-lipid complexes targeting the Tmc1 allele into the cochlea of neonatal Tmc1 Beethoven mice significantly reduced progressive hearing loss 130. The other work led by György et al. further screened 14 Cas9/gRNA combinations for specific and efficient disruption in fibroblasts from Tmc1^Bth/WT^ mice, to improve allele selectivity 131. They failed to use SpCas9 working with a “Near PAM” model, but instead turned to another Cas9 variant, SaCas9-KKH, that recognizes a novel PAM created by the Tmc1 mutation 131. They then packaged AAVs containing SaCas9-KKH and the discriminating sgRNA and delivered them to sensory hair cells of the cochlea. This allele-specific strategy successfully targeted the mutant Tmc1 allele, and restored the hearing sensitivity in Beethoven mice, up to even one-year post injection 131.

Not surprisingly, a variety of other diseases were also studied using this allele-specific strategy **(Table 2)**. For example, György et al. selectively disrupted the APP^sw^ mutation allele both *ex vivo* and *in vivo*, and thereby decreased pathogenic Aβ secretion for treating familial Alzheimer's disease (AD) 132. Christie et al. have tried to use different Cas proteins including SpCas9, SaCas9 and AsCpf1 respectively, based on the diverse mutations of *TGFBI* in treating autosomal dominant corneal dystrophy 133. Wu et al. successfully used the allele-specific CRISPR to help correct a dominant mutation of *CGYGC* gene that causes cataracts, since the stage of mice zygotes 134. The work by Shinkuma et al. successfully applied this strategy to dominant dystrophic epidermolysis bullosa (DDEB), caused by a dominant negative mutation in the *COL7A1* gene encoding type VII collagen in iPSCs 135. And Yamamoto et al. performed an allele-specific ablation that could rescue electrophysiological abnormalities of cardiomyocytes derived from a human iPSC model of long-QT syndrome (LQTS) with a *CALM2* mutation 136. Moreover, Courtney et al. established allele-specific cleavages of the dominant-negative *KRT12* L132P mutated allele, which causes Meesmann's epithelial corneal dystrophy (MECD) 137. Recently, Rabai et al. also examined the allele-specific inactivation or correction of a heterozygous mutation in the *DNM2* gene, which causes the autosomal dominant centronuclear myopathies (CNMs) 138. All in all, the allele-specific strategy has been increasingly implemented, and thus shows tremendous therapeutic potentials for dominantly inherited diseases.

### Allele-specific CRISPR and SNPs Exploitation

Common SNPs representing a given haplotype can all be used for allele-specific targeting of mutant alleles caused by different mutations, which can make allele-specific therapy strategies more versatile and cost-effective **(Figure 3B)**. Recently, two proof-of-principle studies have documented this strategy for treating Huntington's disease (HD) 59, 60. HD is a dominantly inherited neurodegenerative disorder caused by CAG repeat expansions in the huntingtin (*HTT*) gene that results in an elongated polyglutamine (polyQ) tract in the huntingtin protein 139-141. The pathogenic HTT basically consists of more than 35 CAG repeats and produces intracellular polyQ aggregates, preferentially causing the death of medium spiny neurons in the striatum, via a gain of toxic function 142, 143. Deletion of the dominant mutant allele would leave one normal allele intact, which is sufficient for normal function and prevent the onset of the disease. Therefore, they deigned the allele-selective CRISPR/Cas9 strategy hopefully for selectively inactivating mutant HTT allele. They first screened for possible “In PAM” SNPs that are present merely on the mutant but not on normal chromosome haplotype. They then designed two allele-specific sgRNAs (**“In PAM”**) flanking the region of mutant allele 59, 60. A large DNA stretch spanning the promoter region, transcription start site and the CAG repeat mutation, was eventually selectively excised. This excision completely silenced the generation of mutant HTT mRNA and protein in HD primary fibroblasts 59, 60 and later confirmed *in vivo* by AAV injection of discriminating Cas9/sgRNAs into a BacHD mouse model 60. Those results of perfectly allele-specific genome targeting would definitely inspire us to treat dominant disorders like HD, by using SNP based allele-specific personalized therapy.

### Allele-specific CRISPR and Haploinsufficiency

Apart from working on gain-of-function mutations, CRISPR has also been applied to activate specific genes that may be promising for loss-of-function treatments. Loss-of-function mutations in one allele can lead to haploinsufficiency, a condition that reduced protein doses may not be able to assure normal functions and consequently cause human diseases. It is now estimated that there are more than 660 genes associated with human diseases due to haploinsufficiency 144. Although delivering additional copies of the gene via recombinant AAV (rAAV) is an effective and safe gene therapy, it still has some limitations, including limited size of DNA packaging (~5.0 kb, including inverted terminal repeats) and ectopic expression 145. However, increasing the endogenous expression levels of the normal allele could overcome those limitations and potentially correct haploinsufficiency **(Figure 3A)**.

Matharu et al. recently deployed a strategy of CRISPR-mediated activation (CRISPRa) for treating obesity, whereby dCas9 is fused with a transcriptional activator, VP64, so as to target one gene's regulatory element 146. They focused on two genes, *SIM1* and *MC4R*, which are both expressed in the paraventricular nuclei (PVN) of the hypothalamus and are necessary for the regulation of body weight. The haploinsufficiency of SIM1 and MC4R due to loss-of-function mutations would lead to hyperphagic obesity 147. They thus designed discriminating sgRNAs that specifically recognize the SIM1 promoter (Prm-CRISPRa) or its distant hypothalamic enhancer (Enh-CRISPRa) 146. Using the SIM1^+/-^ mice as the obesity model, they observed that both Prm-CRISPRa and Enh-CRISPRa mice showed obviously reduced body fat and food intake 146. Interestingly, the upregulated levels of SIM1 by CRISPRa targeting turned to be in a tissue-specific manner. They only observed significantly elevated mRNA levels in the hypothalamus or kidney, two tissues where SIM1 is expressed, but not in the lung and liver, where the regulatory elements are not active and SMI1 is not expressed 146. This tissue-specificity endows an extra safety feature to the CRISPRa therapeutics.

They further employed rAAV to deliver CRISPRa (with both SpCas9 and SaCas9) into the hypothalamus of SIM1^+/-^ mice or MC4R^+/-^ mice respectively. It gave rise to several fold increase in SIM1 mRNA expression, and similarly rescues the weight gain phenotypes of mice model 146. Those findings demonstrated that the CRISPRa system can rescue a haploinsufficient phenotype *in vivo*. Importantly, this CRISPRa strategy only targets endogenous regulatory elements to enhance the expression of the existing functional genes, without DNA breaks. Therefore, it can overcome the problem of ectopic gene expression, and can also be applied for genes that are not amenable to conventional gene therapy, due to that they have longer coding sequences beyond the rAAV packaging limitations.

Notably, one of the possible side effects after allele-specific targeting in dominant diseases, might be that the remaining normal alleles are causing haploinsufficiency. This would be diseases- and mutations-dependent. For targeting HTT alleles as mentioned above 59, loss of one copy of HTT did not actually generate typical HD symptoms, and one functional copy of HTT is sufficient to maintain normal cellular physiology. And also in the case of KRT12 137, the expression of the dominant-negative mutant L132P is abolished by NHEJ, which could be considered a therapeutic success, as KRT12 has been shown not to demonstrate haploinsufficiency 148. However, for those situations that may cause haploinsufficiency, it might be practical to combine CRISPRa to activate the normal copy simultaneously when selectively targeting of the mutant allele. Additionally, the CRISPR-mediated interference (CRISPRi) could also be employed to target the mutants and thereby suppressing their expression levels **(Figure 3A)**. This CRISPRa/i strategy might contribute a fine-tuned control of expression levels, which may be appended to the current “all-or-none” paradigm of allele-specific targeting.

### Allele-specific CRISPR and Epigenome Editing

In most cases of genome editing, both the paternal and maternal alleles of a given locus are targeted. For cases such as X-chromosome inactivetion and genomic imprinting diseases, however, one of the alleles is silenced due to the CpG methylation. Specifically, genomic imprinting is a unique epigenetic process in mammals, characterized by differential DNA methylation in the parental alleles at imprinting control regions (ICRs) 149, 150. This differential methylation results in a mono-allelic expression of genes (paternal or maternal) without altering the DNA sequence. Genomic imprinting controls the expression of around 100 human genes, which directly or indirectly modulate fetal growth, frequently manifesting as severe developmental and neurological diseases, such as Silver-Russel syndrome, Beckwith-Wiedemann syndrome and Angelman syndrome 151.

Instead of binding directly on methylated DNA strands, epigenetic editing is a powerful way to alter DNA methylation at implicated loci. Allele-specific targeting is thus particularly applicable for treating those disorders. The easy and flexible targeting of the CRISPR-dCas9 system may allow to recruit particular DNA binding proteins/domains, which specifically bind to either allele of an imprinted locus in the patients 83. The polymorphisms between maternal and paternal alleles would confer their discriminations. For example, the dCas9 fused with chromatin-modifying proteins such as DNA methyltransferases DNMT3A or demethylase domains, may facilitate an allele-specific binding of discriminating sgRNA at imprinted loci, and further reverses their methylated status 152, 153 **(Figure 3C)**.

### Allele-specific CRISPR and Immunocompatibility

When implementing cell transplantations, one major concern that must be overcome before clinical application is the potential human leukocyte antigen (HLA) mismatch, which can lead to immune rejection 154. The cell therapies using iPSCs show to be promising in treating a variety of diseases, due to their capabilities of differentiating into a wide range of specialized cell types and tissues. However, it is time-consuming and costly to produce autologous clinical grade iPSCs for individual patients 155. One alternative to personalized iPSC therapy is to establish a bank of clinically grade iPSC lines that can be differentiated for use in a broader number of patients 156. HLA homozygous donors are being considered for covering most HLA haplotypes, however, recruiting HLA homozygous donors for the entire population is challenging. In a recent study, Xu et al. developed tailored HLA-editing strategies to facilitate the immunocompatibility of iPSCs 157. They first generated HLA pseudo-homozygous iPSCs, after the allele-specific editing in HLA heterozygous iPSCs. HLA genes are highly polymorphic, so that the HLA codes of target alleles must be considered in designing the genome editing strategy. Moreover, all HLA genes have sequence similarity, which increases the risk of targeting non-specific HLA alleles. They thus generated a customized gRNA database for the SpCas9 system to involve all of the available HLA haplotype sequences from the IPD-IMGT/HLA database 158. Among the 7,955 gRNAs with an ''NGG'' PAM sequence, they figured out 7,118 gRNAs that can target individual HLA genes, of which only 2,388 are able to target single alleles. They succeeded in generating class I HLA haploid iPSCs by allele-specific HLA targeting of heterozygous cells, thus producing so-called pseudo-homozygous iPSCs, which should have similar donor potential as HLA homozygous iPSCs 157. This strategy could significantly improve donor compatibility in regenerative medicine, and is expected to serve pseudo-homozygous iPSCs for the majority of the world's population **(Figure 3D)**.

## Specificity and Off-Target

### Truncation of sgRNAs

It is believed that there should be a balance between the on-target efficiency and specificity for a given sgRNA, because increasing specificity may also lead to a reduction in on-target cleavage. In terms of genome targeting for therapy, even with the allele-specific method, specificity is a very critical element that must be taken into account. So far, enormous efforts have been made to minimize potential off-target activities.

One of the strategies is the usage of a truncated sgRNA (17-18 nt rather than 20 nt in length), which was demonstrated to improve specificity 159, 160. However, this conclusion appears to be quite controversial in the practice of genome editing by allele-specific CRISPR. Several studies have conducted allele-specific targeting with truncated sgRNAs. Christie et al. used both the mutant 18 and 20 nt sgRNAs for the cleavage of the mutant allele 133. While the truncated 18 nt sgRNAs did not provide obvious improvements of specificity, maximal discrimination occurred with 20 or 19 nt sgRNAs for most cases 133. Budde et al. made genome-editing efforts on correcting patient iPSCs harboring heterozygous dominant mutations that cause FTD 114. They compared the effects of different lengths of allele-specific sgRNAs. A truncated sgRNA (17 nt) produced 6 fold lower NHEJ frequency than the full-length sgRNA (20 nt) and also fewer corrected iPSC clones. Moreover, whole genome sequencing showed that the mutational burden in both corrected iPSCs was similar, demonstrating that truncated sgRNAs at this site do not really enhance allele-specific targeting 114. Similarity was also observed in a recent report by Gao et al. 130. The full-length sgRNA (Tmc1-mut3) modified the mutant Tmc1 allele 23-fold more efficiently than the wild-type one, whereas the corresponding truncated 17 nt sgRNA (Tmc1-mut4) almost diminished its discrimination ability 130. To the contrary, there were also reports echoing this strategy of using truncated sgRNAs. For instance, Li et al. successfully documented a truncated sgRNA (17 nt) that can improve allele discrimination with a cleavage efficiency of 28% of RHO P23H, without detectable cleavages in the wild-type alleles 124.

Those controversial results might be due to the status of the binding energy between RNA and DNA. The truncated gRNA works because the binding energy is reduced enough to bind a perfect target rather than to include mismatched targets 159. Therefore, it reminds us that the usage of truncated gRNAs might be locus-dependent and empirically evaluated.

Beside the truncation of sgRNAs, there are other various approaches with the potential to improve targeting specificity, by using (1) low level or short-term exposure of Cas9: direct delivery of Cas mRNA/proteins 34, 161-163; (2) Cas9 nickase (Cas9n) 164, or the engineered Cas nucleases with increased fidelity (such as SpCas9-HF 46, eSpCas9 47, HypaCas9 48, SaCas9-HF 49 and enAsCpf1-HF1 50); (3) chemically modified sgRNAs 165, 166; (4) controllable Cas9 167, 168; (5) fusions of dCas9 with FokI nuclease domain 169, 170, and so forth. As this topic is not within the scope of this review and has already been well-described elsewhere 81, 171, 172, hence we will not discuss it with much more details. In the preclinical practice, we should try all the best to lower off-target activities of Cas nucleases, probably by the combinatorial approaches to optimize both Cas9 and sgRNA design 173.

### Single-nucleotide Skipping

Another important phenomenon termed single-nucleotide skipping may also intervene with the allele-specific CRISPR. Although it is believed that even single nucleotide difference can confer the discrimination for allele-specific targeting, studies have shown that nucleotide mismatches at certain positions can be tolerated by CRISPR via single-nucleotide skipping 174, 175. When DNA sequence contain single base insertion ("DNA bulge") or deletion ("RNA bulge"), the genomic locus can also be cleaved by Cas9 174.

In another study, frequent off-target were observed in genome sites with a single-base bulge or up to 13 mismatches between the sgRNA and its genomic target 175, suggesting that single-nucleotide skipping from either the sgRNA or its genomic target can be tolerated.

Furthermore, a recent study conducted allele-specific genome editing, using the single G insertion in the p16^ink4a^ gene model, and tested whether a single-nucleotide gap could also be exploited for allele-specific editing by CRISPR 176. Interestingly, they found that the cleavage of target sites caused by single-nucleotide skipping can be observed if the gap occurs at the 1^st^ or 2^nd^ base upstream of the PAM 176. Whereas skipping between the 3^rd^ and 7^th^ bases is unlikely to occur, or to be less effective in tolerating mismatches 176. The general Cas9 tolerated DNA bulges in target sites lied in three regions: 7 bases from PAM, the 5'-end (PAM-distal) and the 3'-end (PAM-proximal) 176. Although more studies are required to determine whether these positions are common for different target sequences, single-base DNA-bulges may be considered during off-target analysis. And also this brings a caveat for manipulating single-base variants in allele-specific targeting, if without an extra positional consideration of the variants.

## Designing Allele-specific CRISPR

### Designing Guidance

**Figure 4:**
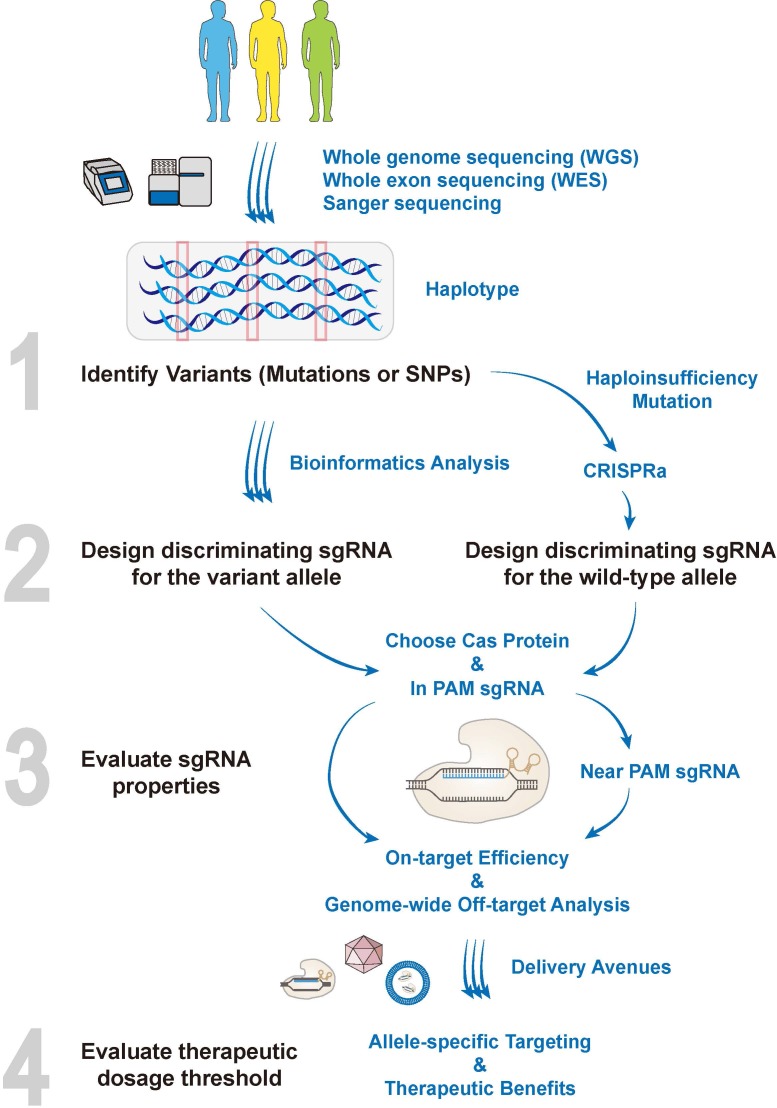
** The pipeline of designing allele-specific CRISPR.** In the era of precision medicine, haplotypes of patients are revealed by kinds of sequencing techniques. The personalized SNPs that represent a given haplotype, as well as genetic mutations, can all be exploited for allele-specific targeting. Bioinformatics tools are then employed for designing discriminating allele-specific sgRNAs combined with the optimal Cas protein. Notably, for genetic variants that may cause haploinsufficiency, it might be practical to combine CRISPRa to activate the wild-type allele simultaneously when selectively targeting of the variant allele. Basically, designing the discriminating sgRNAs needs to examine if (i) genetic variants generate novel PAM sites (In PAM sgRNA); or (ii) genetic variants have PAM sites nearby, placing the variants within the seed region (Near PAM sgRNA). To allow the best discrimination, the “In PAM sgRNA” is the priority recommendation that contributes to the most stringent allele-specific cleavages. The properties of discriminating sgRNAs such as GC contents and base constitutions, would then be taken into the evaluation of their on-target cleavage efficiencies. Delivery avenues such as AAVs, ribonucleoprotein (RNPs) or lipid nanoparticles (LNPs) will be employed to carry the Cas protein and discriminating sgRNAs into targeting sites. Importantly, the safety should be assessed by unbiased whole-genome off-target analysis at the preclinical stage. Finally, the dosage threshold for achieving therapeutic benefits after allele-specific targeting is disease-dependent, which needs to be empirically evaluated.

To edit the genomes in an allele-specific manner, basically an analysis of genetic variants in a given haplotype needs to be performed to examine if (i) they generate novel PAM sites; or (ii) they have PAM sites nearby, placing the variants within the seed region **(Figure 4)**. This echoes the two working models of allele-specific CRSIPR. To allow the best discrimination, the “In PAM” model is the priority recommendation that contributes to the most stringent allele-specific cleavages. While taking a closer look at the discriminating sgRNAs, their features per se such as high/low GC contents, consecutive T bases or self-complementarity, may greatly dampen their on-target cleavage efficiencies 177, 178. Therefore, the combined “In PAM” possibilities and sgRNA properties has to be taken into account. With the increasing choices of engineered Cas and PAM sites, it is worth designing multiple discriminating sgRNAs to be validated, before they are applied to gene therapies **(Figure 4)**.

### Bioinformatics Tools

Figuring out appropriate sgRNAs that may discriminate between two alleles is labor intensive and time consuming 60, 133, whereas not even approaching complete options. To aid the rational design of discriminating sgRNAs, therefore, a robust bioinformatics tool is highly desired **(Figure 4)**. Although numerous tools are now available to assist with sgRNA selection for a reference genome 178, few have been implemented for allele-specific purposes. So far, tools like **AlleleAnalyzer** 113 and **AsCRISPR** 179 were recently developed for allele-specific and personalized sgRNA design. **AlleleAnalyzer** is a software that involves single-nucleotide variants and short indels to design dual sgRNAs for editing one or multiple haplotypes 113. It also leverages patterns of shared genetic variations annotated by the ExAC and 1000GP to optimize sgRNA design for different human populations 64, 113.

It is worth noting that an increasing amount of non-coding RNAs or regulatory elements are identified, dual-sgRNA deletion of a large DNA fragment or gene locus might bring unpredictable risks for gene therapy. Genome targeting with a single allele-specific sgRNA should be an effective alternative strategy. The other tool, **AsCRISPR**, is a web server recently implemented in our lab that can process with either heterozygous allele sequences or Reference SNP cluster IDs, and would like to provide a complete list of potential single allele-specific sgRNAs working with more than 20 Cas proteins 179. It is thus more like a query-driven tool focusing on the research studies and clinical therapeutics for the CRISPR community.

## Therapeutic Perspectives

### NHEJ vs. HDR

Conventional gene therapy shows great promise in the treatment of recessive diseases. However, their use in dominant diseases is limited because of the need for silencing or ablation of gain-of-function or dominant-negative mutant alleles, as well as introducing wild-type copies as replenishments that may even beyond the maximal packaging capacity of AAVs. In situ DNA repair by precise knock-in is an alternative avenue, involving the HDR pathway and a stretch of DNA donor. The low efficiency of HDR by CRISPR (generally less than 10%), however, still represents a practical challenge that hinders its applications 180, 181. One potential advantage of allele-specific CRISPR would be that it merely employed the NHEJ pathway, instead of HDR, for efficiently targeting deleterious alleles. Without the need of providing DNA donors or viral vectors, it may enormously augment the therapeutic efficacy and reduce the complexity and cost of the therapy.

Moreover, HDR only occurs during late S and G2 phases when DNA replications are completed and sister chromatids can serve as repair templates, whereas NHEJ dominates DNA repair during G1, S and G2 phases 182. Therefore, the knock-in method requiring HDR is less suited for post-mitotic cells such as neurons, whereas the allele-specific CRISPR using the NHEJ machinery could circumvent this issue and thus be applied to specifically target neurons in treating neurological diseases.

### Personal Genomes and PAM Constraints

As a quite novel personalized strategy, allele-specific genomic targeting requires a thorough analysis of patients' haplotype patterns. This recapitulates the importance of personal genome sequencing (or genetic diagnosis) in implementing personalized therapy. Allele-specific CRISPR will then be applied to treating individual patients, starting with the identification of suitable polymorphisms that meet the designing criteria **(Figure 4)**.

However, one limitation of the current technique is that it will require a unique variant-derived PAM or a PAM site close enough to the variant of interest. In some cases, there may be no appropriate choices of discriminating sgRNAs, and the cleavage efficiency of candidate sgRNAs might be even compromised. It urgently needs the growing expansion of the CRISPR-Cas toolbox with broader choices of PAM sites. The goal is to re-engineer Cas nucleases for desirable characteristics, including altered PAM sequences, better packaging into virus, better binding and cutting efficacy and higher specificity. And hopefully it can achieve highly efficient and allele-specific knockout of most, if not all, human dominant alleles in the practice of gene therapies.

### Dosage Considerations

As mentioned earlier, allele-specific silencing of one mutant allele in dominant diseases gives rise to a 50% reduction in the expression level of the normal copy, if no compensation happens, and might probably cause haploinsufficiency, depending on the properties of given mutations **(Figure 4)**. This reminds us of the potential dosage considerations during allele-specific targeting. It brings up several critical concerns, before the implementation of genomic engineering, that (i) whether the half amount of proteins from the remaining normal allele suffices to ameliorate or restore a disease phenotype; or vice versa (ii) to which extent would the augmentation/ correction of normal gene expression levels can produce therapeutic benefits in treating haploinsufficiency?

Certain diseases like Hemophilia B (HB), an X-linked genetic bleeding disorder caused by deficiency of coagulator factor IX (*FIX*), can be significantly restored by as low as 0.56% genetic correction 183. Likewise, another study employed CRISPR to correct the dystrophin gene mutation in the germ line of mdx mice, the animal model of Duchenne muscular dystrophy (DMD) 184. They generated several genetically mosaic animals containing 2% to 100% correction of the dystrophin gene. Strikingly, correcting just 17% of the mutant alleles was sufficient to allow dystrophin expression in most myofibers, and the muscles exhibited less histopathological features than mdx muscle 184. The percentage of muscle phenotypic rescue (47% to 60%) in mosaic mice even exceeded the efficiency of gene correction (17%), demonstrating the advantage of corrected cells and their contribution to regenerating muscles 184. Currently, one clinical trial is using SaCas9 and delivery by AAV into the retina to treat Type 10 Leber congenital amaurosis (LCA10), a severe retinal dystrophy caused by mutations in the *CEP290* gene, by removing the aberrant splice donor created by the IVS26 mutation and thereby restoring normal CEP290 expression 185. It has shown that it would be necessary to rescue 10% of foveal cones to achieve clinical benefits that are sufficient for near-normal visual acuity 185.

Importantly, the proliferative advantage of target cells after genome editing may also allow that correcting a low number of cells would be sufficient to reverse the disease phenotypes. For example, in treating patients with Fanconi anemia (FA), NHEJ-mediated gene editing was employed to efficiently edit multiple *FANCA* mutations in long-term hematopoietic stem cells (HSCs) and lymphoblastic cell lines (LCLs) 186. Those corrected FA-HSCs showed significant proliferation advantage and phenotypic correction both *in vitro* and after transplantation *in vivo*, due to that NHEJ was positively employed to generate compensatory therapeutic mutations that may restore *FANCA* functions 186. Specifically, they showed that therapeutic indels were initially at a low ratio (0.20%) and were greatly enriched during proliferation (47.36% at day 60), which successfully restored nuclear FANCD2 foci formation, increased mitomycin C resistance, reduced chromosomal fragility upon diepoxibutane challenge, and reduced reactive oxygen species levels in edited cells 186. This study also echoes that, instead of HDR, NHEJ-based gene targeting could provide a simple therapeutic avenue for treating a specific group of patients with monogenic lympho-hematopoietic diseases.

Altogether, those delightful results may somehow relieve the researchers' anxiety for searching applicable sgRNAs with the highest efficiencies, but meanwhile remind us of a necessary pre-evaluation with the dosage “threshold” of therapeutic effects after a genome editing-based therapy 187. It is also worth noting that the required dosage is quite dependent on given disease or mutation types, therefore, the therapeutic dosage of allele-specific targeting needs to be empirically evaluated.

An extra specific concern is that wild-type allele expressions may also be slightly dampened under less stringent allele-specific cleavages directed by certain discriminating sgRNAs 124, 130. We are trying our best to avoid this, however in some cases, we may have no better choices. Theoretically, the reduction of 5%-10% of normal proteins leading to minor hypofunction might be tolerated in biological systems. Hence, it might be an acceptable trade-off if the dominant pathogenic alleles are potently disrupted.

### Germline Editing and Ethical Dilemma

So far, almost all endeavors on allele-specific genome targeting were achieved either *ex vivo* or *in vivo* in affected somatic tissues. *Ex vivo* approaches are generally restricted to certain cell types that can be edited in the lab and subsequently transplanted, such as HSCs 186. *In vivo* approaches, to the contrary, could be employed to a wider range of tissues, but the potential side effects induced by off-targets still represent a major safety concern. Delightfully, great efforts have been devoting to achieve tissue-specific genome targeting, to lower the risk of possible deleterious off-target indels in non-pathogenic tissues 188, 189.

The somatic gene therapy, however, cannot prevent transmission of mutated genes from parents to offspring, which may persist the entire family's medical burden. As such, it looks like that germline editing in reproductive cells (sperm and eggs) or preimplantation embryos holds greater potential for correcting disease-causing mutations at an early stage that may prevent the inheritance of genetic disease to all future generations. However, genome editing of human embryos is far from mature and will also raise numerous grave safety, social and ethical concerns. One recent study ever tried to edit β-Globin gene in nonviable human embryos, and it turned out that the targeting efficiency was low and, most critically, it brought about substantial off-target effects and mosaicism 190, which is a common issue occurred in germline editing that leads to the mixed untargeted and targeted cells within a multicellular embryo 191. Better technologies for improving targeting specificities, thoroughly detecting off-targets and eliminating mosaicism have to be developed to secure the human applicability of germline editing. However, even with great technical advancements in future, human germline editing may raise other social concerns such as new forms of social inequality and discrimination that still needs to be extensively debated.

## Summary

Overall, incorporating genetic variations into sgRNA designs successfully enabled the allele-specific genome engineering. The strategy of allele-specific CRISPR is now increasingly believed to be promising for treating genetic diseases for individual patient. Allele-specific genomic targeting has been versatile in increasing research areas, including the treatment of dominant negative diseases, genome imprinting, haploinsufficiency, spatiotemporal loci imaging and immunocompatible manipulations. Combined with other emerging tools such as CRISPR base editors, more advanced applications are likely to be exploited. However, more endeavors on improving sgRNA specificities, expanding recognized PAM sites, better packaging into AAVs, better binding and cutting efficacy and safer delivery of Cas nucleases, have to be addressed before it can be applied to humans.

## Figures and Tables

**Table 1:** The CRISPR toolbox for genome DNA engineering

Cas Nucleases	PAM Sequence	PAM Location	Size (aa)	TracrRNA Requirement	Cutting Manner	References	Notes
**Type II System: Cas9 & Variants**							
*SpCas9*	NGG	3' end	1368	Yes	Blunt	19, 20, 33, 34	SpCas9 can also cleave sgRNA target sites followed by 'NAG', however with efficiency reduced to ∼20%
*SpCas9-VRER*	NGCG		1368			51	Has a stringent selectivity for an NGCG PAM sequence
*SpCas9-EQR*	NGAG		1368			51	Specific for an NGAG PAM
*SpCas9-VQR*	NGA		1368			51	Strongly recognizes sequences bearing the NGAN PAM
*SpCas9-VRQR*	NGA		1368			46	Has improved activities relative to the VQR variant on sites with NGAH (H = A, C, or T) PAMs
*FnCas9*	NGG		1629			52	One of the largest Cas9 orthologs; exhibits slight activities toward those with the TGA and TAG PAMs
*FnCas9-RHA*	YG		1629			52	An engineered FnCas9 variant
*NmCas9*	NNNNGHTT		1082			21-25	Preferred consensus PAM (5'-NNNNGATT-3') for NmeCas9 genome editing in human cells; Suitable for AAV package
*Nme2Cas9*	NNNNCC		1082			26	Suitable for AAV package
*GeoCas9*	NNNNCRAA NNNNGMAA		1087			27	A thermostable Cas9
*St1Cas9*	NNAGAAW		1121			22, 28, 51	
*St3Cas9*	NGGNG		1409			28	
*TdCas9*	NAAAAC		1423			22	
*CjCas9*	NNNNACAC NNNNRYAC		984			29-31	Suitable for AAV package
*ScCas9*	NNG		1375			32	
*xCas9*	NG, GAA and GAT		1368			53	Used phage-assisted continuous evolution; an expanded PAM SpCas9 variant that can recognize a broad range of PAM sequences
*SpCas9-NG*	NG		1368			54	
*SaCas9*	NNGRRT		1053			35	Suitable for AAV package
*SaCas9-KKH*	NNNRRT		1053			55	An engineered SaCas9 variant
*cSaCas9*	Multiple PAMs		1053			56	Identified several chimeric SaCas9 variants with expanded recognition capability at NNVRRN, NNVACT, NNVATG, NNVATT, NNVGCT, NNVGTG, and NNVGTT PAM sequences
**Type V-A System: Cpf1 (Cas12a) & Variants**							
*AsCpf1*	TTTV	5' end	1307	No	Staggered	36-38	Has a lower activity at a TTTT PAM. CTTA also led to high indel frequencies for both AsCpf1 and LbCpf1, which may be considered as a secondary PAM, especially for LbCpf1 38
*LbCpf1*	TTTV		1228			36, 38	Has a lower activity at TTTT PAM
*FnCpf1*	TTV		1300			36, 39	May manifest different activities depending on the organisms; KYTV reported in 39
*Mb3Cpf1*	TTV		1261			40	Exhibits comparable activity to AsCpf1 and LbCpf1 with TTTV PAMs; Can recognize a TTV PAM, but with lower efficiency
*AsCpf1-RR*	TYCV		1307			57, 58	Also cleaves ACCC and CCCC PAMs (and, to a lesser extent, VYCV)
*AsCpf1-RVR*	TATV		1307			57, 58	Also cleaves RATR PAMs
enAsCpf1	TTYN VTTV TRTV		1307			50	
ArCpf1 BsCpf1 PrCpf1	TTN		1262 1206 1213			41	
HkCpf1	YTN TYYN		1310			41	
**Type V-B System: Cas12b (C2c1) & Variants**							
*AaCas12b*	TTN	5' end	1129	Yes	Staggered	42	
*AkCas12b*	TTTN		1147			43	
*BhCas12b v4*	ATTN		1108			43	Also works at a subset of TTTN and GTTN PAMs, albeit with less robust activities
**Type V-E System: CasX (Cas12e) & Variants**							
*DpbCasX*	TTCN	5' end	986	Yes	Staggered	44, 45	
*PlmCasX*	TTCN		978			44, 45	

**Table 2:** Summary of studies on disease treatment by allele-specific CRISPR

Targeted Genes	Variants Types	Variants Locations	Cas Nucleases	PAM	Disease Types	Targeting Specificity	Functional Outcomes	References
*RHO*	P23H	-3 nt PAM	SaCas9	NNGRRT	Retinitis pigmentosa (RP)	Indel formation was detected in the mutant His allele only	Delivered to both patient iPSCs *in vitro* and pig retina *in vivo*, and created a frameshift or premature stop that would prevent P23H transcription	122
*RHO*	P23H	-4 nt PAM	SaCas9-KKH	NNNRRT	Retinitis pigmentosa (RP)	No detectable cleavage was found either at WT or P23H allele		123
*RHO*	P23H	-4 nt PAM	SpCas9-VQR	NGA	Retinitis pigmentosa (RP)	Presented a high rate of cleavage in the P23H but not WT allele	Slowed photoreceptor degeneration and improved retinal functions	123
*RHO*	P23H	-12 nt PAM	SaCas9-KKH	NNNRRT	Retinitis pigmentosa (RP)	(i) Unable to distinguish the mutant P23H allele from the wild-type one; (ii) Robust cutting efficiencies of 37.8% were observed in the injected WT mice, even though SaCas9-KHH preferentially targeted the mutant allele		124
*RHO*	P23H	-4 nt PAM	SpCas9-VQR	NGA	Retinitis pigmentosa (RP)	(i) Unable to distinguish the mutant P23H allele from the wild-type one; (ii) Robust cutting efficiencies of 40% were observed in the injected WT mice, even though SpCas9-VQR preferentially targeted the mutant allele; (iii) Truncated sgRNA (17 nt) improved allele discrimination with a cleavage efficiency of 28%, and no detectable cleavage in the WT controls		124
*RHO*	P23H	-4 nt PAM	SpCas9- VRQR	NGA	Retinitis pigmentosa (RP)	Truncated sgRNA (17 nt) paired with SpCas9-VRQR cleaved the P23H allele with greater efficiency (~ 2 fold) compared that with SpCas9-VQR, but also brought about an increase in targeting of the WT allele from 0% to 1.3 ± 0.3%	(i) Significantly delayed progression of photoreceptor cell degeneration in the outer nuclear layer; (ii) The low-level disruption of the WT allele did not abrogate the observed therapeutic benefit	124
*RHO*	S334ter	Novel PAM	SpCas9	NGG	Retinitis pigmentosa (RP)	No cleavage was detected at the RHO WT allele	Prevented retinal degeneration and improved visual function in rat model	125
*KRAS*	G13A	Novel PAM	SpCas9	NGG	Colorectal cancer	Completely silenced the mutant allele; No aberrant effects on the WT allele	Reversal of drug resistance to the MEK inhibitor	126
*EGFR*	L858R	Novel PAM	SpCas9	NGG	Non-small cell lung cancer (NSCLC)	Small indels were detected in the EGFR mutant allele with a frequency of 3.6% (± 0.1%) at 2 days post-transfection; no mutations were detectably induced in the WT allele	Enhanced cancer cell killing and inhibition of tumor growth	127
*BRAF*	V600E	+1 nt PAM	As/LbCpf1	TTTN	Melanoma	The efficiency of AsCpf1 was very weak, and no activity was detected using LbCpf1		128
*BRAF*	V600E	+13 nt PAM	As/LbCpf1	TTTN	Melanoma	Both AsCpf1 and LbCpf1 show robust cleavage activities only in mutant sequence		128
*BRAF*	V600E	-11 nt PAM	SpCas9	NGG	Melanoma	SpCas9 cut the mutant allele ~ 4-fold more efficiently than the WT allele		128
*BRAF*	V600E	Novel PAM	SpCas9-EQR	NGAG	Melanoma	No cleavage events were unexpectedly observed by Cas9-EQR for both WT and mutant allele		128
*TMC1*	M412K	-6 nt PAM	SpCas9	NGG	Hearing loss	Modified the mutant TMC1 allele 23-fold more efficiently than the WT allele. Edited the WT TMC1 locus much less efficiently (0.066-1.6% indels). A truncated sgRNA decreased indel % on the mutant allele and further dampened its discrimination ability	Reduced progressive hearing loss and improved acoustic startle response	130
*TMC1*	M412K	Novel PAM	SaCas9-KKH	NNNRRT	Hearing loss	Indel formation only in the Tmc1 allele; very little (0.0075%) in the WT one	Injected mice exhibit normal or near-normal thresholds of auditory brainstem responses	131
*APP*	KM670/671NL (APPswe)	-1/-2 nt PAM	SpCas9	NGG	Early-Onset Alzheimer's Disease (EOAD)	(i) CRISPR-induced indels were only detected in APP^SW^ alleles but not in APP^WT^ alleles after deep sequencing detection; (ii) A truncated sgRNA (17 nt) was inefficient in disrupting the APP^SW^ allele	Decreased the secretion of Aβ40 and Aβ42	132
*TGFBI*	L527R	Novel PAM	SpCas9	NGG	Corneal dystrophy	(i) Only resulted in cleavage of the mutant reporter; the WT reporter remained intact; (ii) sgRNA truncation did not improve specificity		133
*TGFBI*	R555W	Novel PAM	SaCas9	NNGRRT	Corneal dystrophy	Unable to distinguish between WT (NNGRRC) and mutant TGFBI (NNGRRT) sequence, due to the comparable efficiencies of recognizing NNGRRT/V		133
*TGFBI*	R124L	Novel PAM	AsCpf1	VYCV	Corneal dystrophy	Can distinguish between WT and mutant TGFBI sequence, but with a low efficiency		133
*TGFBI*	R124C, R124H, R124L, R555Q, R555W	Differ by a single base pair in the spacer	SpCas9	NGG	Corneal dystrophy	(i) Cut WT alleles with varying efficiencies; (ii) Truncated sgRNAs did not provide marked improvements of specificity, for most cases, maximal discrimination occurred with 20 or 19 nt guides; (iii) The additional G at the 5' end of the guide sequence did not provide an improved specificity in any case		133
*CRYGC*	Leu160Stop	Novel PAM	SpCas9	NGG	Nuclear cataracts	No gene editing events in the WT allele	The targeted mutant allele repaired by HDR	134
*COL7A1*	c.8068_8084delinsGA	Short Indels: Different base pairs since -5 nt PAM	SpCas9	NGG	Dystrophic epidermolysis bullosa (DDEB)	Specifically targeted only the mutant sequence of COL7A1	Edited COL7 degraded at the protein level and could not undergo collagen triple helix formation	135
*CALM2*	N98S	-1 nt PAM	SpCas9n	NGG	Early-onset long-QT syndrome (LQTS)	Obtained 7 of 18 clones with mutant allele-specific genome modification, and another 7 clones with both WT and mutant allele targeted. Hard to tell the specificity, due to the Cas9 double nickase system containing both upstream WT sgRNA and downstream mutant-specific sgRNA	Rescued the abnormal electrophysiological properties of iPSC derived cardiomyocytes	136
*KRT12*	L132P	Novel PAM	SpCas9	NGG	Meesmann's epithelial corneal dystrophy (MECD)	No effects on the WT allele	Achieved both *in vitro* and *in vivo* KRT12 mutation-specific targeting	137
*DNM2*	R465W	-1 nt PAM	SpCas9	NGG	Centronuclear myopathies (CNMs)	Used 18 bp truncated gRNAs. Analysis of single-cell clones showed 60% NHEJ in diseased fibroblasts. All of the NHEJ events detected occurred exclusively on the mutated allele	Targeting the mutated allele ameliorated disease-related phenotypes including the alterations in endocytosis and transferrin uptake, as well as autophagy defects	138
*HTT*	mHTT (CAG180, CAG69)	Novel PAM (derived from the promoter and intron 3)	SpCas9	NGG	Huntington's disease (HD)	The combined usage of two allele-specific gRNAs selectively excised ~44 kb DNA spanning promoter region, transcription start site, and CAG expansion mutation of HTT, resulting in complete inactivation of the mutant allele without affecting the normal one	Completely prevented the generation of mHTT mRNAs and proteins	59
*HTT*	mHTT (CAG57, CAG97)	Novel PAM (derived from the promoter)	SpCas9	NGG	Huntington's disease (HD)	Targeted only on the PAM-containing mutant allele	Reduced mHTT expressions in both primary fibroblast and BacHD transgenic mice	60

## Abbreviations

1000GP: 1000 Genomes Project
AAV: adeno-associated virus
AD: Alzheimer's disease
adRP: autosomal dominant retinitis pigmentosa
ASOs: antisense oligonucleotides
Cas9n: cas9 nickase
CNMs: centronuclear myopathies
CRISPR: clustered regularly interspaced short palindromic repeats
CRISPRa: CRISPR-mediated activation
CRISPRi: CRISPR-mediated interference
DDEB: dominant dystrophic epidermolysis bullosa
DMD: Duchenne muscular dystrophy
DSBs: double-strand breaks
dsDNA: double strand DNA
ExAC: Exome Aggregation Consortium
FA: Fanconi anemia
FIX: factor IX
FTD: frontotemporal dementia
gnomAD: Genome Aggregation Database
GWAS: genome-wide association studies
HB: hemophilia B
HD: Huntington's disease
HDR: homology-directed repair
HLA: human leukocyte antigen
HSCs: hematopoietic stem cells
HTT: huntingtin
ICRs: imprinting control regions
iPSCs: induced pluripotent stem cells
LCA10: Type 10 Leber congenital amaurosis
LCLs: lymphoblastic cell lines
LncRNA: long noncoding RNA
LQTS: long-QT syndrome
MECD: Meesmann's epithelial corneal dystrophy
NHEJ: non-homology end joining
NSCLC: non-small cell lung cancer
PAM: protospacer adjacent motif
polyQ: polyglutamine
PVN: paraventricular nuclei
RHO: rhodopsin
sgRNA: single guide RNA
siRNAs: short-interfering RNAs
SNPs: single nucleotide polymorphisms
TALEN: transcription activator-like effector nucleases
